# Right Heart Contrast Echocardiography Microbubble Count and Migraine Severity: A Dose–Effect Relationship Study

**DOI:** 10.1002/clc.70155

**Published:** 2025-06-02

**Authors:** Haijuan Gu, Wenjun Fan, Jiesheng Xia, Jianwei Shi

**Affiliations:** ^1^ Department of Ultrasound Haimen District People's Hospital Nantong China; ^2^ Department of Cardiology Haimen District People's Hospital Nantong China

**Keywords:** migraine, patent foramen ovale, right heart contrast echocardiography

## Abstract

**Objective:**

This study aimed to investigate whether a dose–effect relationship exists between the number of microbubbles detected on right heart contrast echocardiography (RHCE) and the clinical severity of migraine.

**Methods:**

We conducted a cross‐sectional study of 190 adult patients diagnosed with migraine who underwent RHCE. Microbubble counts were categorized into four grades per frame (Grades 0–III) based on their appearance in the left atrium within three to six cardiac cycles after right atrial opacification. Migraine severity was assessed using the Migraine Disability Assessment (MIDAS) score and the Headache Impact Test (HIT‐6). Multivariate linear regression was used to evaluate the association between microbubble grades and migraine severity. The predictive ability of the model was assessed using the residual plots and variance inflation factors. Sensitivity analyses were performed to test the robustness of the findings by adjusting for potential confounders.

**Results:**

A clear dose–response relationship was identified, with patients in higher microbubble‐grade groups demonstrating significantly elevated MIDAS and HIT‐6 scores (*p* < 0.001). Patients with Grade III microbubbles reported the highest mean MIDAS (18.2 ± 6.1) and HIT‐6 (64.8 ± 4.9) scores, compared to those in lower grades (*p* < 0.001). Regression analyses confirmed that the higher microbubble burden independently predicted migraine severity (*β* = 0.46, *p* < 0.001). Sensitivity analyses yielded consistent findings.

**Conclusion:**

Our results suggest a notable dose–effect relationship between RHCE microbubble count and migraine severity. These findings highlight the potential role of right‐to‐left shunting as a physiological contributor to migraine.

## Graphical abstract

We conducted a cross‐sectional study of 190 adult patients diagnosed with migraine (with or without aura) who underwent RHCE for suspected right‐to‐left shunt. Our results suggest a notable dose–effect relationship between RHCE microbubble count and migraine severity. These findings highlight the potential role of right‐to‐left shunting as a physiological contributor to migraine.

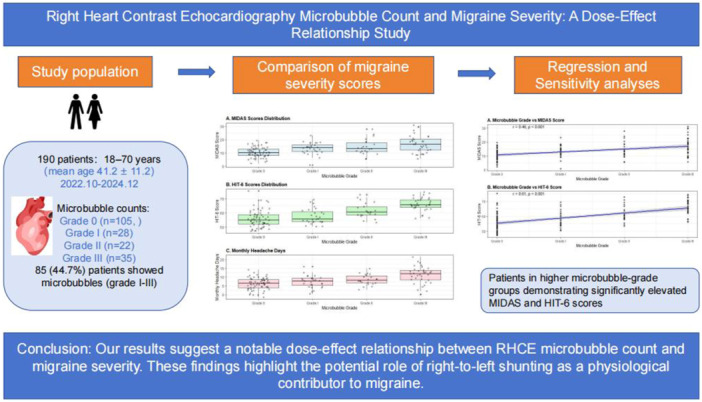

## 1 Introduction

Migraine is a prevalent neurological disorder affecting an estimated 15%–20% of the global population, with a female predominance [1, 2]. It is characterized by recurring headache attacks that are often unilateral, pulsating, and exacerbated by routine physical activity, frequently accompanied by nausea, vomiting, photophobia, or phonophobia [3]. Accumulating evidence has suggested that structural or functional right‐to‐left cardiac shunts, commonly identified as a patent foramen ovale (PFO), may be implicated in migraine pathogenesis or modulation [4, 5, 6]. This right‐to‐left shunt could, in some individuals, trigger or worsen migraine attacks through paradoxical embolism or by transporting vasoactive mediators that instigate cortical or meningeal irritation [7]. The link between PFO and migraine is strengthened by clinical observations that certain individuals with migraine, particularly those with aura, may experience a reduction in migraine burden after percutaneous closure of a demonstrable PFO [8, 9]. Nevertheless, the consistency of this therapeutic effect remains a matter of ongoing debate [10].

Right heart contrast echocardiography (RHCE) is a vital, noninvasive (or minimally invasive) diagnostic modality for detecting right‐to‐left shunts, such as a PFO. Typically, RHCE involves the injection of saline or gel‐based microbubbles into a peripheral vein while performing echocardiography [11]. Traditionally, the characterization of PFO presence has been described qualitatively or semi‐quantitatively (e.g., small, moderate, or large shunt). More recently, quantitative or more refined semi‐quantitative methods have emerged, aiming to capture the actual volume of shunted blood or the number of microbubbles crossing the atrial septum [12]. While numerous studies have examined PFO presence versus absence in the migraine population, considerably fewer have attempted to explore the dose–effect relationship—that is, whether the magnitude of right‐to‐left shunting correlates with the severity of migraine [13]. Establishing the presence of a dose–effect relationship could yield novel insights into the pathophysiology of migraine and facilitate more personalized approaches to management, including the consideration of PFO closure in carefully selected individuals [14].

This study aimed to use RHCE to detect and grade microbubble passage in migraine patients, examining whether the count of microbubbles observed in the left atrium correlates with the severity of their migraine headaches. The clinical implications of these findings, and future directions for research that might pave the way for targeted interventions.

## 2 Methods

### 2.1 Study Design and Participants

We conducted a cross‐sectional observational study at a tertiary academic medical center between October 2022 and December 2024. The study was approved by the local institutional ethics committee, and all participants provided written informed consent before enrollment. The inclusion criteria were: (1) age 18–70 years; (2) clinical diagnosis of migraine (with or without aura) based on the International Classification of Headache Disorders, 3rd edition (ICHD‐3) criteria [15]; and (3) referral for RHCE due to suspicion of right‐to‐left shunt, typically for investigating cryptogenic stroke risk or migraine with suspected cardiac etiologies.

Patients were excluded if they had (1) incomplete RHCE studies or technical difficulties precluding reliable quantification of microbubbles; (2) a known intracardiac defect other than PFO (e.g., atrial septal defect) that was already repaired; (3) significant valvular heart disease or heart failure (NYHA Class III–IV); (4) a history of prior cardiac surgery (e.g., congenital corrections, valve replacements) which could confound the results; or (5) concurrent severe neurological or psychiatric illnesses that would impede reliable migraine severity measurement.

### 2.2 Right Heart Contrast Echocardiography Protocol

All echocardiographic studies were performed by two experienced cardiologists, each with more than 10 years of echocardiography experience. A commercially available echocardiogram machine (Siemens SC2000 or Philips EPIQ 7C) was used for standard transthoracic imaging. The contrast medium comprised saline agitated with a small volume of air using a two‐syringe method connected by a three‐way stopcock to create microbubbles [16].

With patients in the left or right lateral decubitus position, baseline standard 2D echocardiography was conducted to evaluate cardiac anatomy and function. Then, 10 mL of the saline contrast solution was rapidly injected into a peripheral vein (right antecubital vein). The appearance of microbubbles in the right atrium was observed in real time. Subsequently, we focused on the detection of any microbubbles passing into the left atrium. The entire procedure was recorded digitally.

To standardize quantification, each patient performed a Valsalva maneuver or a sniff test during contrast injection, as previously recommended to increase right atrial pressure and facilitate right‐to‐left shunt detection [17]. Microbubble counts were evaluated during the first three to six cardiac cycles after right atrial opacification. We adopted a four‐grade classification of microbubble passage in the left atrium: Grade 0: no microbubbles observed; Grade I: 1–10 microbubbles; Grade II: 11–30 microbubbles; Grade III: more than 30 microbubbles (or a “shower” of bubbles). All echocardiograms were independently evaluated by two cardiologists who were blinded to the clinical data. Discrepancies in grading were resolved by consensus.

### 2.3 Clinical Assessment of Migraine

Migraine severity was ascertained by two validated instruments: the Migraine Disability Assessment (MIDAS) questionnaire and the Headache Impact Test (HIT‐6).

MIDAS was assessed using a 5‐item questionnaire evaluating the number of days of missed activities in the previous 3 months, including work or school, household work, and social or leisure activities [18]. Higher MIDAS scores indicate greater migraine‐related disability. The MIDAS scale has demonstrated good reliability and validity in previous studies, and its scores are correlated with clinicians' judgment regarding the need for medical intervention [18, 19].

The HIT‐6 was utilized to evaluate the broader impact of headaches on patients' quality of life. HIT‐6 includes six questions covering domains such as social role functioning, pain severity, emotional well‐being, cognitive function, and vitality [20]. Scores range from 36 to 78, with higher scores indicating a greater impact of headache on daily activities. Specifically, scores of 36–49 suggest little or no impact, 50–55 indicate moderate impact, 56–59 reflect substantial impact, and scores ≥ 60 suggest severe impact on quality of life. The HIT‐6 scale has also demonstrated good reliability and validity, and it is strongly correlated with headache severity [21, 22].

Participants completed both scales at the time of their echocardiography appointment. We also collected data on headache frequency (headache days per month), usage of acute and preventive migraine therapies, and presence or absence of aura from medical records and interviews.

### 2.4 Covariates and Data Collection

Demographic and clinical data included age, sex, body mass index (BMI), smoking status, blood pressure, medication history, and comorbidities such as hypertension, diabetes mellitus, hyperlipidemia, anxiety, or depression. Blood pressure was measured using an automated sphygmomanometer after 5 min of rest. Laboratory data included basic metabolic panels to rule out major electrolyte abnormalities. We also recorded any history of ischemic stroke or transient ischemic attack (TIA), as many referrals were initially for cryptogenic stroke evaluation.

### 2.5 Statistical Analysis

All statistical analyses were performed using SPSS version 26.0 (IBM, Chicago, IL, USA) and R statistical software (version 4.2.1). For descriptive statistics, continuous variables were expressed as mean ± standard deviation (SD) if normally distributed, or median (interquartile range) if non‐normally distributed. Categorical variables were reported as frequencies and percentages. Group comparisons were made using one‐way ANOVA or the Kruskal–Wallis test for continuous variables, as appropriate, and the *χ* ^2^ test for categorical data.

We first conducted unadjusted analyses to evaluate differences in MIDAS and HIT‐6 scores across the four microbubble‐grade groups (Grades 0, I, II, III). Post hoc pairwise comparisons were performed using the Bonferroni correction for multiple testing. For the primary analysis, we constructed a multivariable linear regression model to examine whether microbubble grade independently predicted migraine severity (MIDAS and HIT‐6 as separate outcomes). We incorporated covariates that could plausibly affect migraine severity, including age, sex, BMI, blood pressure, smoking status, presence of aura, and comorbid conditions (hypertension, diabetes, anxiety, and depression). Interaction terms (e.g., microbubble grade × aura) were tested and retained in the model if statistically significant.

We expressed the regression results as *β* coefficients (standardized regression coefficients) with corresponding *p* values and 95% confidence intervals (CIs). An *α* level of 0.05 was used to determine statistical significance. Model diagnostics (residual plots, variance inflation factors) were used to ensure that assumptions of linearity and normality were met.

For sensitivity analyses, we repeated the regression excluding patients on chronic preventive migraine medication (e.g., beta‐blockers, topiramate) since these might influence headache frequency or severity, and we also excluded patients with a prior history of ischemic stroke or TIA. We hypothesized that excluding these subpopulations might reduce potential confounding and clarify the relationship between shunt burden and migraine.

Finally, we performed a trend test (Cochran–Armitage test for trend or Jonckheere–Terpstra test, as appropriate) to assess whether there was an ordered relationship between microbubble grade and migraine severity measures.

## 3 Results

### 3.1 Baseline Characteristics

**Figure 1:**
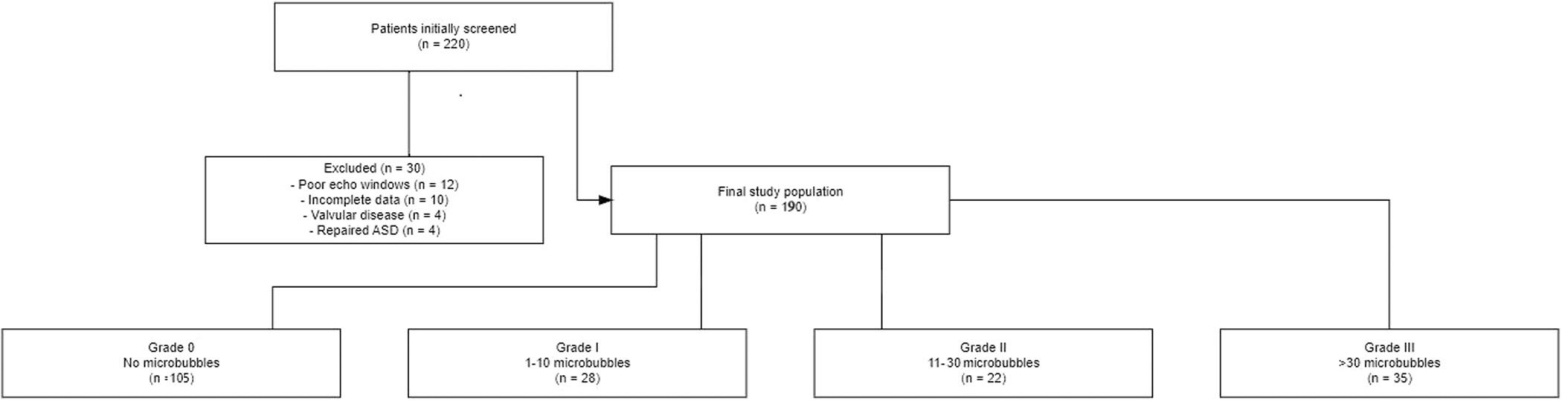
Flow diagram of patient selection and categorization.

A total of 220 patients were initially considered for the study. Of these, 30 were excluded due to poor echocardiographic windows (*n* = 12), incomplete migraine assessment data (*n* = 10), significant valvular disease (*n* = 4), or previously repaired atrial septal defect (*n* = 4). The flow diagram of patient selection and categorization is illustrated in Figure 1. This yielded 190 patients for the final analysis, comprising 142 women (74.7%) and 48 men (25.3%), with a mean age of 41.2 ± 11.2 years. The average duration of migraine was 8.2 ± 5.1 years, and migraine with aura was reported in 76 (40.0%) patients.

Of these 190 patients, 85 (44.7%) exhibited microbubbles in the left atrium (Grades I–III). Specifically, 28 (14.7%) were classified as Grade I, 22 (11.6%) as Grade II, and 35 (18.4%) as Grade III. The remaining 105 (55.3%) displayed Grade 0 (no microbubbles). Table 1 summarizes the key demographic and clinical characteristics stratified by microbubble grade.

**Table 1:** Baseline characteristics by microbubble grade.

Variable	Grade 0 (*n* = 105)	Grade I (*n* = 28)	Grade II (*n* = 22)	Grade III (*n* = 35)	*p*
Age (years)	41.6 ± 10.8	40.9 ± 9.7	40.2 ± 10.3	41.1 ± 11.6	0.72
Female, *n* (%)	77 (73.3)	20 (71.4)	17 (77.3)	28 (80.0)	0.71
BMI (kg/m^2^)	24.4 ± 3.2	24.6 ± 3.4	25.0 ± 3.1	24.8 ± 3.0	0.54
Migraine with aura, *n* (%)	34 (32.4)	12 (42.9)	11 (50.0)	19 (54.3)	0.08
Hypertension, *n* (%)	27 (25.7)	8 (28.6)	5 (22.7)	9 (25.7)	0.93
Diabetes, *n* (%)	10 (9.5)	3 (10.7)	2 (9.1)	3 (8.6)	0.98
Smoking, *n* (%)	24 (22.9)	7 (25.0)	5 (22.7)	8 (22.9)	0.99
Anxiety/Depression, *n* (%)	20 (19.0)	6 (21.4)	5 (22.7)	7 (20.0)	0.94
Stroke/TIA history, *n* (%)	6 (5.7)	2 (7.1)	1 (4.5)	3 (8.6)	0.88

Abbreviations: BMI, body mass index; TIA, transient ischemic attacks.

Detailed analysis of migraine characteristics revealed significant differences across microbubble grades, as shown in Table 2. Notably, patients with higher microbubble grades experienced more frequent headaches and associated symptoms.

**Table 2:** Migraine characteristics by microbubble grade.

Characteristic	Grade 0 (*n* = 105)	Grade I (*n* = 28)	Grade II (*n* = 22)	Grade III (*n* = 35)	*p*
Headache days/month	6.2 ± 3.1	7.8 ± 3.4	9.1 ± 3.8	11.3 ± 4.2	< 0.001
Duration (h)	12.4 ± 6.2	14.1 ± 6.8	15.8 ± 7.1	17.2 ± 7.4	0.002
Photophobia (%)	52.3	60.7	68.2	77.1	0.004
Phonophobia (%)	48.6	57.1	63.6	71.4	0.006
Nausea (%)	46.2	53.6	59.1	68.6	0.005

MIDAS and HIT‐6 scores showed a clear ascending trend with increasing microbubble grade: MIDAS scores: Grade 0: 10.4 ± 4.2; Grade I: 12.6 ± 5.1; Grade II: 15.1 ± 5.8; Grade III: 18.2 ± 6.1 (overall *p* < 0.001). HIT‐6 scores: Grade 0: 55.3 ± 6.2; Grade I: 58.1 ± 5.8; Grade II: 61.2 ± 5.1; Grade III: 64.8 ± 4.9 (overall *p* < 0.001). These relationships are visually represented in Figure 1, which demonstrates the progressive increase in both MIDAS and HIT‐6 scores with higher microbubble grades.

#### 3.1.1 Correlation Analysis

**Figure 2:**
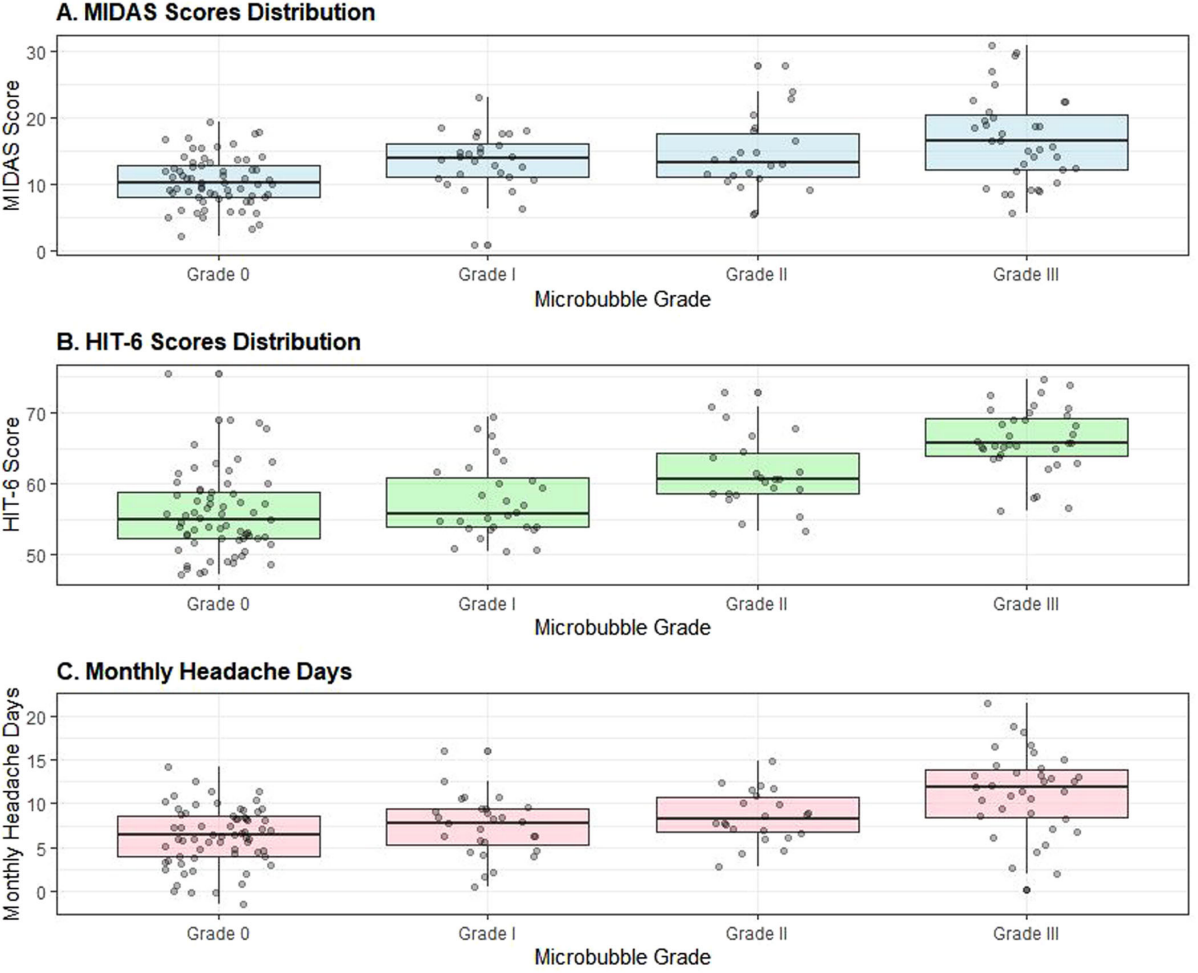
Migraine severity measures across microbubble grades. (A) MIDAS scores distribution. (B) HIT‐6 scores distribution. (C) Monthly headache days. All measures show significant positive correlation with microbubble grade (*p* < 0.001).

Further analysis revealed strong correlations between microbubble grade and migraine severity measures, as illustrated in Figure 2. The correlation coefficients were *r* = 0.52 (*p* < 0.001) for MIDAS scores and *r* = 0.56 (*p* < 0.001) for HIT‐6 scores.

#### 3.1.2 Multivariable Analysis

Multivariable linear regression analysis confirmed that microbubble grade remained an independent predictor of migraine severity after adjusting for potential confounders (Table 3).

**Table 3:** Multivariable linear regression analysis of factors associated with migraine severity.

Variable	MIDAS score *β* (95% CI)	*p*	HIT‐6 score *β* (95% CI)	*p*
Microbubble Grade	0.42 (0.31 to 0.53)	< 0.001	0.46 (0.35 to 0.57)	< 0.001
Female Sex	0.18 (0.06 to 0.30)	0.003	0.19 (0.07 to 0.31)	0.002
Age	0.05 (−0.06 to 0.16)	0.375	0.04 (−0.07 to 0.15)	0.468
BMI	0.03 (−0.08 to 0.14)	0.594	0.02 (−0.09 to 0.13)	0.721
Migraine with Aura	0.16 (0.04 to 0.28)	0.008	0.17 (0.05 to 0.29)	0.006
Hypertension	0.07 (−0.04 to 0.18)	0.211	0.06 (−0.05 to 0.17)	0.285
Smoking	0.04 (−0.07 to 0.15)	0.478	0.05 (−0.06 to 0.16)	0.374
Anxiety/Depression	0.08 (−0.03 to 0.19)	0.158	0.09 (−0.02 to 0.20)	0.108

*Note: R* ^2^ = 0.34 (MIDAS), 0.36 (HIT‐6), adjusted *R* ^2^ = 0.31 (MIDAS), 0.33 (HIT‐6).

Abbreviations: BMI, body mass index; HIT‐6, Headache Impact Test; MIDAS, Migraine Disability Assessment Score.

The model explained approximately one‐third of the variance in migraine severity (adjusted *R* ^2^ = 0.31 for MIDAS and 0.33 for HIT‐6). Microbubble grade demonstrated the strongest association with both MIDAS (*β* = 0.42, *p* < 0.001) and HIT‐6 (*β* = 0.46, *p* < 0.001) scores, followed by female sex and presence of aura.

#### 3.1.3 Sensitivity Analyses

**Figure 3:**
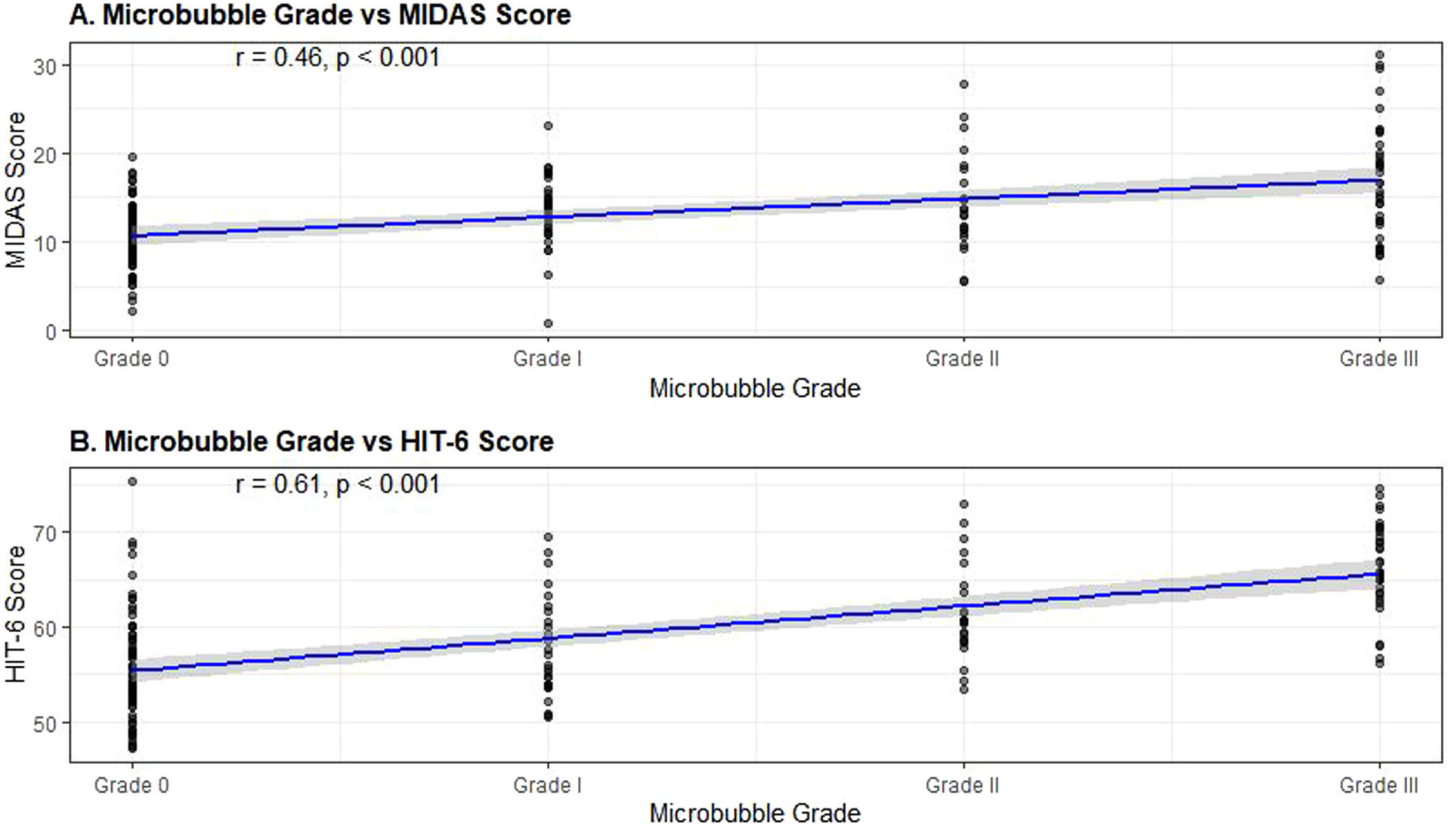
Migraine severity measures across microbubble grades. (A) MIDAS scores distribution. (B) HIT‐6 scores distribution. All measures show significant positive correlation with microbubble grade (*p* < 0.001).

When excluding the 28 patients on regular migraine preventive therapy, the relationship between microbubble grade and migraine severity remained significant (*β* = 0.45, *p* < 0.001 for MIDAS; *β* = 0.49, *p* < 0.001 for HIT‐6). Similarly, excluding the ten patients with prior stroke/TIA did not substantially alter the associations (*β* = 0.43, *p* < 0.001 and *β* = 0.44, *p* < 0.001, respectively) (Figure 3).

The Cochran–Armitage trend test confirmed a significant monotonic increase in migraine severity with escalating microbubble grade (*p* for trend < 0.001), supporting the existence of a dose–effect relationship between right‐to‐left shunt size and migraine severity.

## 4 Discussion

This cross‐sectional study demonstrated a significant dose–effect relationship between the count of microbubbles observed in RHCE and migraine severity, as measured by MIDAS and HIT‐6. Patients with a higher microbubble burden reported more disabling headaches, suggesting a potential pathogenic role of a more pronounced right‐to‐left shunt in migraine expression. These findings encourage a nuanced approach to the evaluation and management of migraines in individuals with suspected or confirmed PFO.

The pathophysiological mechanisms linking PFO or right‐to‐left shunt with migraine have not been fully elucidated, but several plausible pathways have been proposed [23]. One theory posits that microemboli, normally filtered by the pulmonary circulation, may bypass the lung vasculature and enter cerebral arteries, provoking cortical spreading depression and subsequent migraine attacks [7]. Another explanation involves vasoactive or inflammatory mediators (e.g., serotonin, endothelin‐1) that, instead of being metabolized within the lungs, cross directly into systemic circulation and trigger or exacerbate migraine episodes. Our study demonstrated that higher microbubble loads were associated with more severe migraines, suggesting that not only the presence but also the functional extent of the right‐to‐left shunt may play a critical role in migraine severity. Larger or more patent shunts presumably allow more frequent or substantial passage of triggers into the cerebral circulation, amplifying the migraine burden.

An alternative perspective is that the association might reflect an epiphenomenon, with some individuals possessing a genetic or developmental predisposition to both right‐to‐left shunt and migraine. However, the robust dose–effect gradient observed here suggests a more direct physiological link. Further, the presence of a potential “threshold” phenomenon for migraine induction is congruent with the notion that a critical volume or frequency of shunted substances is necessary to precipitate or intensify headache.

These findings bear several clinical implications. First, they provide a potential rationale for the observed interpatient variability in migraine expression among those with a PFO. Clinicians may consider that not all PFOs are equal: the functional significance depends, at least partly, on the size of the right‐to‐left shunt. Second, in patients with medically refractory migraine and a detected PFO, quantifying shunt size through RHCE may help stratify candidates for interventions such as percutaneous closure. Although evidence from randomized controlled trials remains inconclusive regarding the general efficacy of PFO closure for migraine relief, patients with larger functional shunts might derive a more pronounced benefit [8, 10].

Moreover, if future prospective studies confirm that the reduction or elimination of microbubble passage correlates with improved migraine outcomes, it would strengthen the argument for more individualized management. Current guidelines are cautious about recommending closure solely for migraine because of the procedural risks and uncertain long‐term advantages [24]. Yet, in a subgroup with robustly documented large shunts and severe, treatment‐refractory migraine, closure might be contemplated more seriously. Importantly, the present study was not designed to assess the impact of closure on migraine severity; prospective interventional trials would be needed to address that question conclusively.

Previous investigations that examined the link between PFO and migraine frequently employed a binary classification (PFO present or absent), showing that migraine prevalence or severity is higher in PFO‐positive individuals [4, 5, 8]. Fewer studies have delved into the quantitative shunt burden, typically measured via transcranial Doppler (TCD) or traditional transthoracic echocardiography [12, 13]. Our findings are congruent with those of Gollion et al., who suggested a relationship between massive right‐to‐left shunt and migraine severity; however, sample sizes and definitions of “massive” varied across studies [25]. Our standardized four‐grade approach, aligning with established echocardiographic methods, adds methodological rigor to the field.

Additionally, the presence of migraine aura has sometimes been more strongly associated with PFO, but data remain inconsistent [6, 26]. In our cohort, aura was a significant predictor of higher migraine severity in general, but the effect size was modest, and the interaction between microbubble grade and aura status was not significant. This finding may simply reflect insufficient power for a subgroup effect or may indicate that aura is only a partial proxy for more complex pathophysiological underpinnings.

A major strength of our study lies in the relatively large sample size of patients undergoing RHCE, along with the standardized method for grading microbubble passage. The use of two validated migraine severity scales (MIDAS and HIT‐6) enhances the clinical relevance of the findings. Furthermore, we performed a comprehensive multivariate adjustment and sensitivity analyses to account for possible confounders such as medication usage and history of stroke/TIA, yielding robust and consistent results.

Nevertheless, several limitations should be acknowledged. First, the cross‐sectional design precludes causal inference; higher microbubble counts may reflect, rather than cause, more severe migraines. Second, we did not include TCD or transesophageal echocardiography (TEE) data, which could have provided more detailed confirmation of PFO characteristics. Third, despite adjusting for covariates, residual confounding factors such as migraine triggers cannot be completely ruled out. Fourth, as participants were referred for suspected right‐to‐left shunt, the findings may not be generalizable to all migraine patients. Future prospective studies are needed to assess dynamic changes in microbubble counts and their relationship with migraine progression and treatment response. Interventional trials targeting patients with large shunts and investigations into relevant biomarkers may further elucidate the underlying mechanisms.

## Author Contributions

Jianwei Shi conceived of the study and helped to draft the manuscript. Haijuan Gu, Wenjun Fan, and Jiesheng Xia participated in study design, data analysis, and statistics. All authors read and approved the final manuscript.

## Ethics Statement

This study was conducted in accordance with the Declaration of Helsinki and approved by the Ethics Committee of Haimen District People's Hospital.

## Consent

Written informed consent was obtained from all participants.

## Conflicts of Interest

The authors declare no conflicts of interest.

## Data Availability

All data generated or analyzed during this study are included in this article. Further enquiries can be directed to the corresponding author.
